# Ail Proteins of *Yersinia pestis* and *Y. pseudotuberculosis* Have Different Cell Binding and Invasion Activities

**DOI:** 10.1371/journal.pone.0083621

**Published:** 2013-12-27

**Authors:** Tiffany M. Tsang, Jeffrey S. Wiese, Suleyman Felek, Malte Kronshage, Eric S. Krukonis

**Affiliations:** 1 Department of Microbiology and Immunology, University of Michigan School of Medicine Ann Arbor, Michigan, United States of America; 2 Department of Biomedical and Diagnostic Sciences, University of Detroit Mercy School of Dentistry, Detroit, Michigan, United States of America; 3 Department of Biologic and Materials Sciences, University of Michigan School of Dentistry, Ann Arbor, Michigan, United States of America; Institut de Pharmacologie et de Biologie Structurale, France

## Abstract

The *Yersinia pestis* adhesin Ail mediates host cell binding and facilitates delivery of cytotoxic Yop proteins. Ail from *Y. pestis* and *Y. pseudotuberculosis* is identical except for one or two amino acids at positions 43 and 126 depending on the *Y. pseudotuberculosis* strain. Ail from *Y. pseudotuberculosis* strain YPIII has been reported to lack host cell binding ability, thus we sought to determine which amino acid difference(s) are responsible for the difference in cell adhesion. *Y. pseudotuberculosis* YPIII Ail expressed in *Escherichia coli* bound host cells, albeit at ∼50% the capacity of *Y. pestis* Ail. *Y. pestis* Ail single mutants, Ail-E43D and Ail-F126V, both have decreased adhesion and invasion in *E. coli* when compared to wild-type *Y. pestis* Ail. *Y. pseudotuberculosis* YPIII Ail also had decreased binding to the Ail substrate fibronectin, relative to *Y. pestis* Ail in *E. coli*. When expressed in *Y. pestis*, there was a 30–50% decrease in adhesion and invasion depending on the substitution. Ail-mediated Yop delivery by both *Y. pestis* Ail and *Y. pseudotuberculosis* Ail were similar when expressed in *Y. pestis*, with only Ail-F126V giving a statistically significant reduction in Yop delivery of 25%. In contrast to results in *E. coli* and *Y. pestis*, expression of Ail in *Y. pseudotuberculosis* led to no measurable adhesion or invasion, suggesting the longer LPS of *Y. pseudotuberculosis* interferes with Ail cell-binding activity. Thus, host context affects the binding activities of Ail and both *Y. pestis* and *Y. pseudotuberculosis* Ail can mediate cell binding, cell invasion and facilitate Yop delivery.

## Introduction

There are three *Yersinia* species pathogenic for humans. While the enteric pathogens *Y. enterocolitica* and *Y. pseudotuberculosis* cause primarily self-limiting gastroenteritis, and in some cases mesenteric lymphadenitis, *Y. pestis* causes the rapidly fatal disease plague [1], [2]. While *Y. enterocolitica* and *Y. pseudotuberculosis* both cause enteric infections via an oral route of infection, *Y. pseudotuberculosis* and *Y. pestis* are more closely related genetically, estimated to have evolved from one another between 1500 and 20,000 years ago [3].

All three pathogenic *Yersinia* species harbor a virulence plasmid that encodes cytotoxic Yop proteins and the Type III Secretion System (T3SS) required for their delivery to host cells [2]. This process requires adhesion of *Yersinia* to host cells [4], [5], [6], [7]. Adhesins can bind host cells directly or via bridging molecules such as extracellular matrix components [8], [9], [10], [11], [12], [13]. Pathogenic *Yersinia* species produce many adhesins including invasin (Inv) [14], YadA [10], [15], [16], [17], [18], plasminogen activator (Pla) [19], pH 6 antigen (Psa) [20], and Ail [6], [21], [22].

Ail from *Y. enterocolitica* has been well characterized and many functions have been elucidated, including serum resistance and adhesion to and invasion into host cells [23], [24], [25], [26], [27], [28]. *Y. enterocolitica* Ail binds cultured cells in a species-specific manner with adhesion to CHO and HEp-2 cells, but not MDCK cells [23]. Further studies identified Ail point mutants with intermediate and severe serum sensitivity that also affected invasion [29]. In particular, an aspartic acid (D67) and valine (V68) at the C-terminal end of loop 2 were required for both Ail functions.

Ail from *Y. pseudotuberculosis* is reported to lack adhesion and invasion activities when expressed in *E. coli*, although *E. coli* expressing Ail was still able to confer serum resistance [30] due to C4bp and factor H binding, like *Y. enterocolitica*, [31], [32]. Thus, *Y. pseudotuberculosis* Ail has long been believed to have no adhesion capacity.

Ail from *Y. pestis* has been shown to mediate serum resistance [22], [33], auto-aggregation [22], and cell adhesion [6], [22]. Additionally, Ail is a key *Y. pestis* adhesin for Yop delivery and virulence [6], [34]. The extracellular matrix (ECM) proteins fibronectin and laminin are substrates for *Y. pestis* Ail and these Ail-ECM interactions are important for adhesion to host cells and Yop delivery [12], [13], [35]. The crystal structure of Ail from *Y. pestis* has been determined [13] and it belongs to the OmpX family of proteins described as having a flattened β-barrel with four extracellular loops extending above the surface of the bacteria [36], [37]. The four extracellular loops of *Y. pestis* Ail contain 10–21 amino acids each.

Since Ail is a critical molecule for *Y. pestis* adhesion, Yop delivery, and virulence, we wanted to identify residues of Ail required for adhesion and Yop delivery. Although the previous studies of OmpX and *Y. enterocolitica* Ail are useful, the functions of these proteins cannot be translated to *Y. pestis* Ail, as even *Y. enterocolitica* Ail is only 26–80% identical to *Y. pestis* Ail within the four extracellular loops. *Y. pseudotuberculosis* Ail is 98.9–100% identical to *Y. pestis* Ail, depending on the *Y. pseudotuberculosis* strain, with only two amino acid changes, E43D and F126V in the most divergent *Y. pseudotuberculosis* derivatives. As *Y. pseudotuberculosis* YPIII Ail was previously reported to lack cell binding activity [30], we hypothesized these two amino acids should be important for the adhesive activity of Ail. In this study, we revisited the adhesion capacity of Y. *pseudotuberculosis* Ail and observed reduced but significant binding to cultured host cells, relative to *Y. pestis* Ail. Single mutations based on *Y. pseudotuberculosis* Ail introduced into the *Y. pestis* Ail molecule were analyzed for their cell adhesion, cell invasion and Yop delivery functions. *Y. pseudotuberculosis* YPIII Ail and single mutations were defective for host cell binding and invasion by up to 75%, relative to *Y. pestis* Ail, when expressed in *E. coli* and *Y. pestis*. However, the strongest defect observed for Yop delivery was 25%, and most forms had no decrease in Yop delivery relative to *Y. pestis* Ail.

## Materials and Methods

### Strains and Culture Conditions

Bacterial strains and plasmids used in this study are listed in Table S1. *Y. pestis* strains were cultivated in heart infusion broth (HIB) overnight or on heart infusion agar (HIA) for 48 hours at 28°C. *Y. pseudotuberculosis* strains were cultivated in brain heart infusion broth (BHI) overnight or on brain heart infusion agar for 48 hours at 28°C. *E. coli* strains were cultured in lysogeny broth (LB) or LB agar at 28°C or 37°C. Antibiotics were used at the following concentrations for *E. coli* and *Y. pestis*: chloramphenicol (Cm), 25 µg/ml; and ampicillin (Amp), 100 µg/ml. For *Y. pseudotuberculosis* strains, antibiotic concentrations were: kanamycin (Kan), 6 µg/ml; chloramphenicol (Cm), 5 µg/ml, tetracycline (Tet), 2.5 µg/ml; and ampicillin (Amp), 100 µg/ml. Isopropyl-ß-D-thiogalactopyranoside (IPTG) was used at a 100 µM concentration for *E. coli* and *Y. pestis* and 500 µM for *Y. pseudotuberculosis*, unless otherwise noted. The *Y. pseudotuberculosis* strain YPIII was obtained from Dr. Ralph Isberg and YPIII and IP2666 from Dr. James Bliska.

HEp-2 cells were cultured at 5% CO_2_ (37°C) in modified Eagle’s medium (MEM) (Gibco) supplemented with 10% (v/v) fetal bovine serum (FBS) (Gibco), 1% sodium pyruvate (Gibco), and 1% non-essential amino acids (Gibco).

*Y. pestis* strain KIM5 D27 Δ*ail*Δ*pla* was constructed as described previously for KIM5-3001 Δ*ail*Δ*pla* using lambda-RED recombineering [34].

### Construction of Ail-expressing Plasmids

pSK-*ail* Bluescript encoding wild-type *Y. pestis* Ail was constructed by PCR amplification of the *ail* locus from *Y. pestis* strain KIM5 [6] using primers 5′-GCGCGGATCCTTGGCTGGCCACTTTAGTCT-3′ and 5′-GCGCCTGCAGGGTTAGGAGGACGTTAGAAC-3′. The *ail* PCR product was digested with *Bam*HI and *Pst*I and ligated into *Bam*HI/*Pst*I-cut pSK Bluescript (Stratagene). *ail* from the *Y. pseudotuberculosis* strains YPIII and IP2666 were similarly PCR amplified and ligated into pSK Bluescript. All clones were also moved into the IPTG-inducible vectors pMMB207 [38] and pMMB66EH [39] for cell binding studies.

### Generation of Mutations

PCR-mutagenesis was performed using the enzyme Pfu (Stratagene) and primer pairs encoding the mutations Ail-E43D and Ail-F126V. The primers used were complementary to one another and are listed here: ail-E43D top strand 5′ - caaagtcgtgtcaagGACgatgggtacaagttgg and bottom strand 5′ - ccaacttgtacccatcGTCcttgacacgactttg; ail-F126V top strand 5′ - catggaaaggctaaaGTTtcctcaatatttggtc and bottom strand 5′ - gaccaaatattgaggaAACtttagcctttccatg. Ail-T7I/E43D/F126V was generated using pSK-*ail*-E43D/F126V as a template and T7I primers, T7I TOP 5′-tttttatgaataagaTattactggtctcttc-3′ and T7I BOTTOM 5′-gaagagaccagtaatAtcttattcataaaaa-3′. Following PCR amplification using a pSK-*ail* Bluescript-derived plasmid as a template, the PCR reactions were digested with *Dpn*I to cut the template DNA and transformed into DH5alpha [40]. Potential mutant clones were sequenced to confirm that only the target site was mutated and a *Bam*HI*/Pst*I fragment containing the entire open reading frame and ribosome-binding site, was liberated, purified and ligated into the IPTG-inducible plasmid pMMB207 (Cm^R^) or pMMB66EH (Amp^R^).

### Adhesion Assays and Invasion

Adhesion assays were performed as described previously [12], unless otherwise indicated. HEp-2 cells were cultured in 24-well tissue culture plates until reaching 100% confluence. Bacteria at the proper dilution was added to cultured HEp-2 cells at a multiplicity of infection (MOI) of 50∶1. After 1 hour 45 minutes incubation at 37°C in 5% CO_2_ cell-associated bacteria were liberated by the addition of sterile H_2_O containing 0.1% Triton X-100 for 10–20 min. Percent adhesion was calculated by dividing bound CFU by total bacteria in the well and then multiplying by 100. For *E. coli* and *Y. pestis* adhesion, strains were grown overnight in LB or HIB respectively, and then diluted 1∶50 or 1∶10 respectively, into fresh media with 100 µM IPTG for 3 hrs. For *Y. pseudotuberculosis* adhesion, strains were grown overnight in BHI in the presence of 500 µM IPTG, and then diluted 1∶10 into fresh media with 500 µM IPTG for 3 hrs.

Invasion assays were performed similarly, except that at the end of 1 hour 45 minute of bacterial binding, cells were washed once with phosphate-buffered saline (PBS) to remove unbound bacteria and minimal essential medium containing 7.5 µg/ml gentamicin was added for 1 hour at 37°C in 5% CO_2_ to kill extracellular bacteria. Cells were then washed twice with PBS and lysed and plated as described for the adhesion assay. For *E. coli* invasion, strains were grown overnight in LB, then diluted 1∶50 into fresh media with 100 µM IPTG for 3 hrs. For *Y. pestis* and *Y. pseudotuberculosis* invasion assays, strains were induced overnight with 100 µM or 500 µM IPTG, respectively. Then cells were diluted 1∶10 and grown for an additional 3 hrs in the continued presence of 100 µM or 500 µM IPTG prior to addition to cells.

### Bacterial Binding to Fibronectin

Bacterial binding assays were performed as described previously [12]. Briefly, bacterial cells were diluted and added to immobilized fibronectin (Sigma, F2006) and allowed to bind at 37°C for 2 hours. Bacteria bound to the wells were stained with 0.01% crystal violet. After washing away excess crystal violet, the bacterial-associated crystal violet stain was solubilized with an 80%methanol/20%acetone solution. The absorbance was measured at ABS_595_.

### Cytotoxicity Assay

Cytotoxicity assays were performed as described previously [12]. Briefly, *Y. pestis* KIM5 derivatives were added to HEp-2 cells at an MOI of 10. After 4 hours of incubation at 37°C in 5% CO_2_, cells were fixed with 0.5 ml methanol and stained with 0.76 mg/ml Geimsa stain. Rounding was observed and pictures were taken under a phase-contrast microscope. Cytotoxicity was enumerated by counting total cells and the number of round dark purple (shrunken cytoplasm) cells experiencing cytotoxicity in three microscopic fields (∼125 cells/field). Percent cytotoxicity was calculated by dividing rounded cells by total cells. This experiment was performed three times in duplicate, with two fields of cells enumerated/well n = 12. Statistical significance was assessed using the student t test.

### Western Blot Assays

Cultures of bacteria normalized for their OD_600_ (*E. coli* and *Y. pseudotuberculosis*) or OD_620_ (*Y. pestis*) were resuspended in Laemmli sample buffer. Bacterial cell extracts were boiled and run on a 15% SDS-polyacrylamide gel electrophoresis (PAGE) gel. Proteins were either visualized by Coomassie Brilliant Blue straining or transferred to nitrocellulose and probed with rabbit anti-Ail serum (a kind gift from Dr. Ralph Isberg, [30]) at a 1∶500 dilution.

## Results

### Amino Acid Sequence of Ail from Various *Y. pseudotuberculosis* Strains

**Figure 1:**
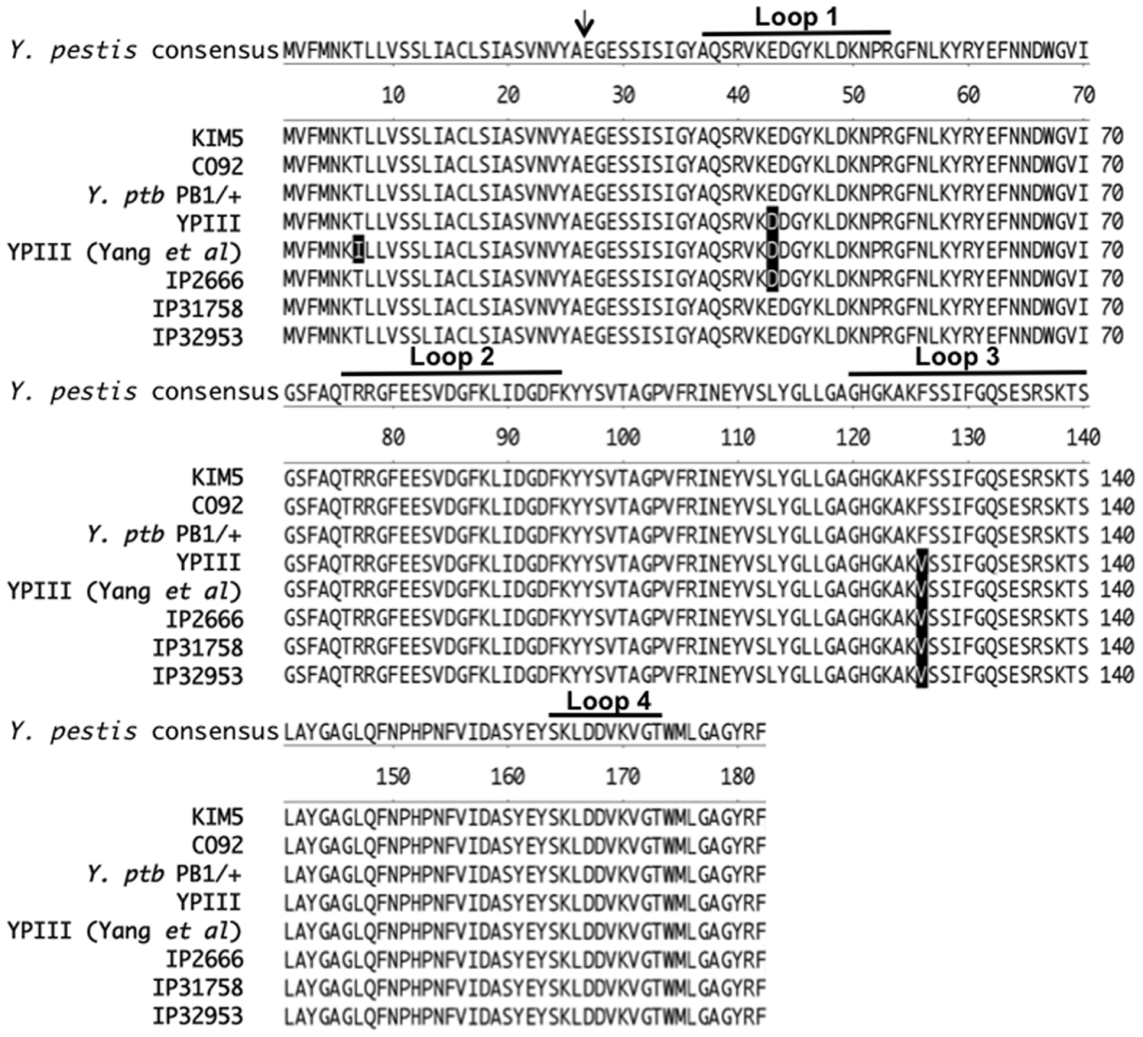
Protein alignment of the various Ail proteins. Ail from *Y. pestis* strains KIM5 ands CO92 and five *Y. pseudotuberculosis* isolates, IP2666 and YPIII, are shown. The two variant amino acids; positions 43 and 126 are highlighted. The arrow indicates the predicted processed cleavage site. The putative extracellular loop regions are indicated with black lines. The alignment was performed with the MegAlign program from Lasergene. KIM5, YPIII and IP2666 *ail* were sequenced in our laboratory. PB1/+, IP31758 and IP32953 *ail* sequences were obtained from genome sequencing projects. YPIII (Yang *et al*) refers to the YPIII sequence reported previously [30].

Prior to determining the relative binding capacities of *Y. pestis* and *Y. pseudotuberculosis* Ail, we compared Ail sequences from representative strains of both *Yersinia* species. For *Y. pestis*, sequences from strains KIM5 and CO92 were used and for *Y. pseudotuberculosis*, sequences strains YPIII, IP2666, PB1/+, IP31758 and IP32953 were used. The sequence alignment (Fig. 1) shows *Y. pseudotuberculosis* YPIII and IP2666 Ail has an aspartate at position 43, instead of a glutamate acid (E43D) and a valine at position 126 instead of a phenylalanine (F126V). The E43D substitution is predicted to lie in surface exposed loop 1 while F126V is predicted to lie in loop 3. At these positions, these two residues may directly contact cell components such as fibronectin [12], [35]. Unlike a previously published YPIII Ail sequence [30], our *Y. pseudotuberculosis* YPIII Ail sequence did not contain an additional amino acid change at position 7, T7I. Analysis from other *Y. pseudotuberculosis* sequencing projects demonstrated that *Y. pseudotuberculosis* strains IP31758 and IP32953 only contain the F126V change relative to *Y. pestis* Ail, and *Y. pseudotuberculosis* strain PB1/+, has an Ail sequence identical to *Y. pestis* (Fig. 1).

### Ail from *Y. pestis* and *Y. pseudotuberculosis* Exhibit Adhesion to Host Cells when Expressed in *E. coli*

**Figure 2:**
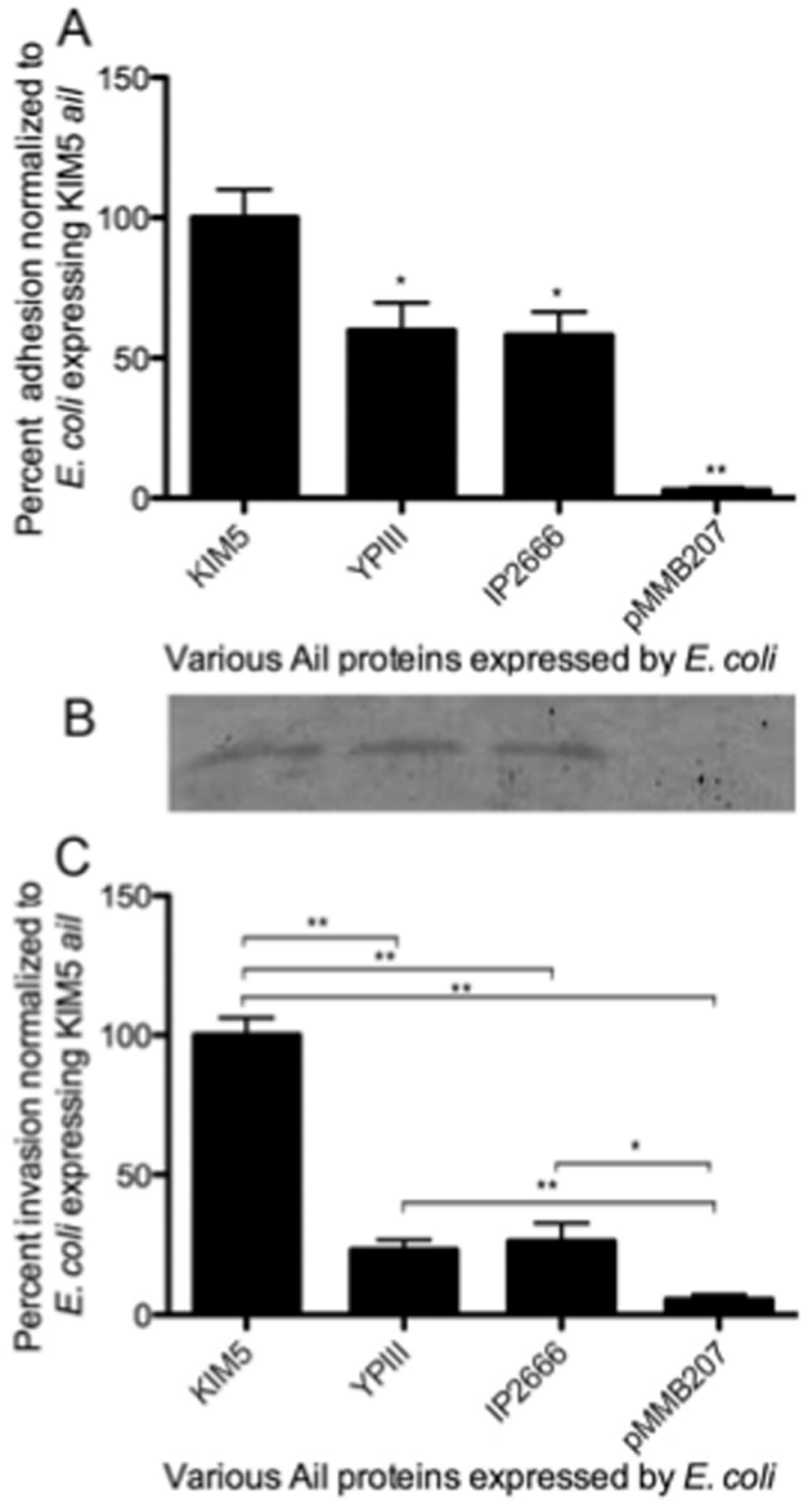
*Y. pseudotuberculosis* Ail exhibits reduced adhesion and invasion function in *E. coli*. (A) Cultured HEp-2 cells were infected with *E. coli* AAEC185 expressing various forms of Ail from the indicated *Yersinia* strains. The empty vector (pMMB207) serves as a negative control. Percent adhesion was calculated by dividing the number of cell-associated CFU by the total number of bacteria in the well and multiplying by 100. The adhesion of *E. coli* AAEC185 expressing KIM5 Ail was set to 100% (actual adhesion was 4.0%). (B) Ail expression levels were determined in whole cell extracts were separated by SDS-PAGE followed by anti-Ail Western blotting. (C) Invasion assays were performed similarly to adhesion assays except infected cells were treated with gentamicin to kill external bacteria. Percent invasion was normalized to 100% for *E. coli*+KIM5 Ail (actual invasion was ∼0.05%). Data are from two independent experiments performed in triplicate (n = 6). *p<0.02; **p<0.00002. Significance was calculated using the Student t test.

To determine the relative binding efficiencies of Ail proteins from different *Yersinia* strains to host cells, Ail from the *Y. pestis* strain KIM5 [6], *Y. pseudotuberculosis* YPIII and *Y. pseudotuberculosis* IP2666 were expressed from an IPTG-inducible construct in *E. coli* AAEC185 (a non-fimbriated strain of *E. coli*, [41]), and adhesion to cultured HEp-2 cells was measured. Adhesion of *E. coli* expressing KIM5 Ail to HEp-2 cells was set to 100% (actual adhesion was 4.0%). In contrast to a previous report [30], we observed significant adhesion activity from the two *Y. pseudotuberculosis* alleles (both encode identical ORFs, Fig. 1). Ail from the two *Y. pseudotuberculosis* strains exhibited ∼60% adhesion to HEp-2 cells, relative to *Y. pestis* Ail, while *E. coli* harboring the pMMB207 empty vector had ∼2% adhesion relative to *Y. pestis* Ail (Fig. 2A). Whole cell lysates demonstrated *E. coli* expressing the various Ail constructs made similar amounts of Ail protein (Fig. 2B). These data indicate that while *Y. pseudotuberculosis* Ail has reduced adhesion ability relative to *Y. pestis* Ail when expressed in *E. coli*, it does mediate adhesion to cultured cells *in vitro*.

The various Ail proteins were also tested for their ability to facilitate *E. coli* invasion of HEp-2 cells, an activity previously reported for *Y. enterocolitica* [23], [42], and *Y. pestis* Ail [22]. While KIM5 Ail protein mediates low levels of invasion in this system (0.01%), this level is 50-fold above background *E. coli* invasion. Relative to KIM5 Ail-mediated invasion (set at 100%), Ail from *Y. pseudotuberculosis* YPIII and IP2666 had 25% invasion activity. *E. coli* expressing vector alone invaded with only 2% of the efficiency of KIM5 Ail (Fig. 2C). These results indicate that like adhesion function, invasion activity of *Y. pseudotuberculosis* YPIII Ail was significantly lower than *Y. pestis* Ail when expressed in *E. coli*.

### Single *Y. pseudotuberculosis*-like Amino Acid Changes within *Y. pestis* Ail also Confer Reduced Adhesion and Invasion Function

**Figure 3:**
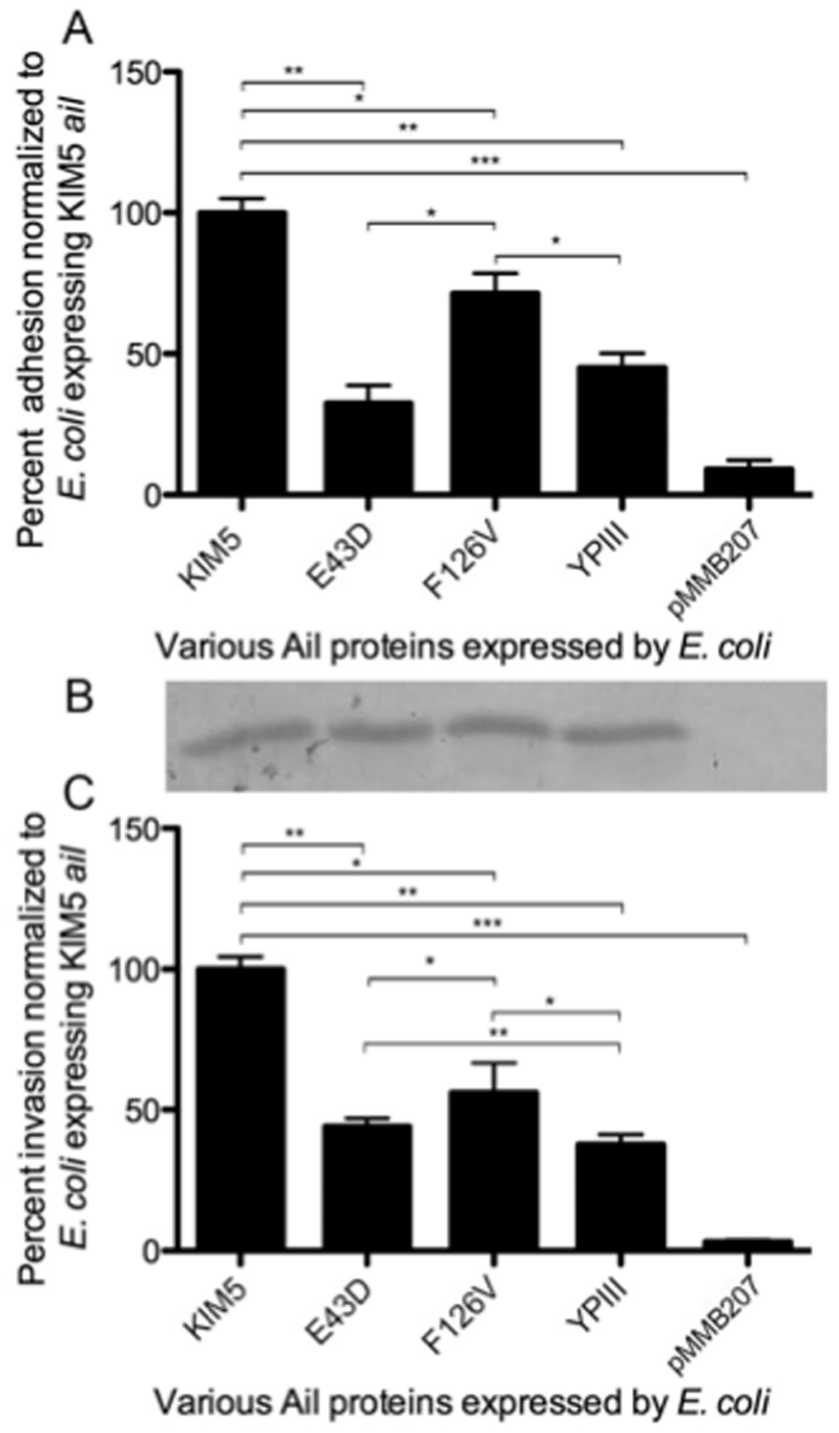
*Y. pestis* Ail single mutants, E43D and F126V, have decreased adhesion and invasion ability in *E. coli*. (A) HEp-2 cells were infected with *E. coli* AAEC185 expressing the *Y. pestis* KIM5 Ail, single mutants of *Y. pestis* Ail, *Y. pseudotuberculosis* YPIII Ail (two substitutions relative to *Y. pestis* Ail), and vector alone. Percent adhesion was calculated by dividing the number of cell-associated CFU by the total number of bacteria in the well and multiplying by 100. HEp-2 adhesion average = 4.7%. (B) Ail expression levels were determined in whole cell extracts separated by SDS-PAGE followed by anti-Ail Western blotting. (C) Invasion assay were performed similar to adhesion assay except infected cells were treated with gentamicin to kill external bacteria. The level of KIM5 Ail-mediated invasion was 0.05% prior to normalization. Data are from two independent experiments performed in triplicate (n = 6). *p<0.03; **p<10^−6^; ***p<10^−9^. Significance was calculated using the Student t test.

We demonstrated that Ail from *Y. pseudotuberculosis* strains YPIII and IP2666 have reduced adhesive activity relative to *Y. pestis* Ail (Fig. 2A). Thus, we wanted to determine which of the two amino acid differences contributed to the reduced adhesion ability. We generated each of the single amino acid mutant derivatives, Ail-E43D and Ail-F126V, and tested their ability to mediate *E. coli* binding to HEp-2 cells. Adhesion to HEp-2 cells was normalized to the wild-type *Y. pestis* (KIM5) Ail construct. The Ail-E43D mutant exhibited about 32% adhesion when compared to KIM5 Ail, while the Ail-F126V mutant gave about 71% adhesion (Fig. 3A). Like previous results (Fig. 2A), *Y. pseudotuberculosis* YPIII Ail mediated 50% adhesion relative to *Y. pestis* Ail. Again, the expression of various Ail derivatives was analyzed by Western blotting and the levels of protein were comparable across all strains (Fig. 3B).

Invasion function of the single amino acid mutants in the KIM5 Ail protein was also assessed. The invasion capacity of the two single mutants were similarly reduced as the Ail-E43D and Ail-F126V mutations gave 45% and 55% invasion, respectively. For both adhesion and invasion, the Ail-E43D mutant had significantly less activity than the Ail-F126V mutant. We presume the slightly increased level of *Y. pseudotuberculosis* YPIII Ail-mediated invasion (45%) as compared to previous experiments (Fig. 2C) is due to experimental variation.

### KIM5 Ail and YPIII Ail Bind to Purified Fibronectin with Different Efficiencies

**Figure 4:**
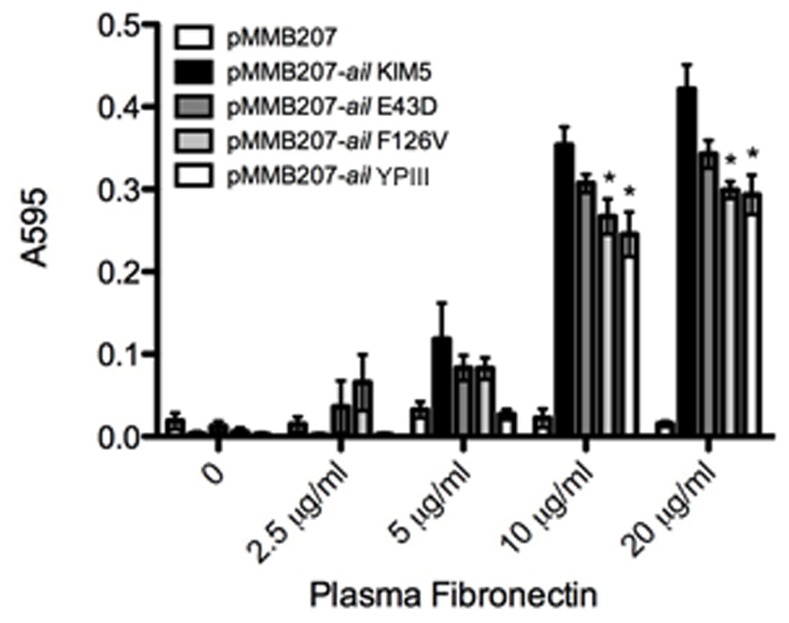
*Y. pseudotuberculosis* YPIII Ail is attenuated for binding to purified fibronectin relative to *Y. pestis* Ail. Purified plasma fibronectin was immobilized on 96-well plates. *E. coli* AAEC185 derivatives expressing the indicated Ail derivatives were added to wells and allowed to bind at 37°C. Bound bacteria were stained with 0.01% crystal violet. Stained bacterial cells were solubilized and the plates were read at ABS_595_. Shown is representative data from two independent experiments done in triplicate (n = 6). *p<0.05 compared to *E. coli* expressing pMMB207-*ail* KIM5.

We have previously shown that fibronectin is a host cell substrate for *Y. pestis* Ail [12]. Therefore, we determined whether reduced binding of *Y. pseudotuberculosis* YPIII/IP2666 Ail to purified fibronectin could account for the difference in host cell binding. *E. coli* expressing various Ail derivatives were added to increasing concentrations of purified plasma fibronectin coated on microtiter plates. *E. coli* expressing KIM5 Ail on its surface bound to fibronectin in a manner that neared saturation as concentrations approached 20 µg/ml (Fig. 4). *E. coli* expressing the Ail-E43D, Ail-F126V, and *Y. pseudotuberculosis* YPIII/IP2666 Ail bound fibronectin less efficiency than *Y. pestis* Ail at coating concentrations of 10 µg/ml and 20 µg/ml. These decreases were significant for Ail-F126V (p = 0.02) and *Y. pseudotuberculosis* YPIII Ail (p = 0.03). Ail-E43D showed a trend toward reduced binding but did not reach statistical significance (p = 0.08). There was also a trend towards reduced fibronectin binding for Ail-E43D, Ail-F126V and YPIII Ail at 5 µg/ml, but those differences did not reach statistical significance (for *Y. pestis* Ail vs. YPIII Ail, p = 0.11). Thus, the various Ail proteins have similar binding patterns to purified fibronectin, but KIM5 Ail appears to bind fibronectin slightly more efficiently than the other derivatives tested.

### Defects in Adhesion and Invasion Activity of Various Ail Derivatives are Maintained in *Y. pestis*

*Y. pestis* has distinct surface characteristics compared to *E. coli* strains such as AAEC185 owing to differences in their LPS core structure [43]. Thus, we determined whether our various Ail derivatives had a defect in adhesion when expressed in the more natural context of *Y. pestis*.

**Figure 5:**
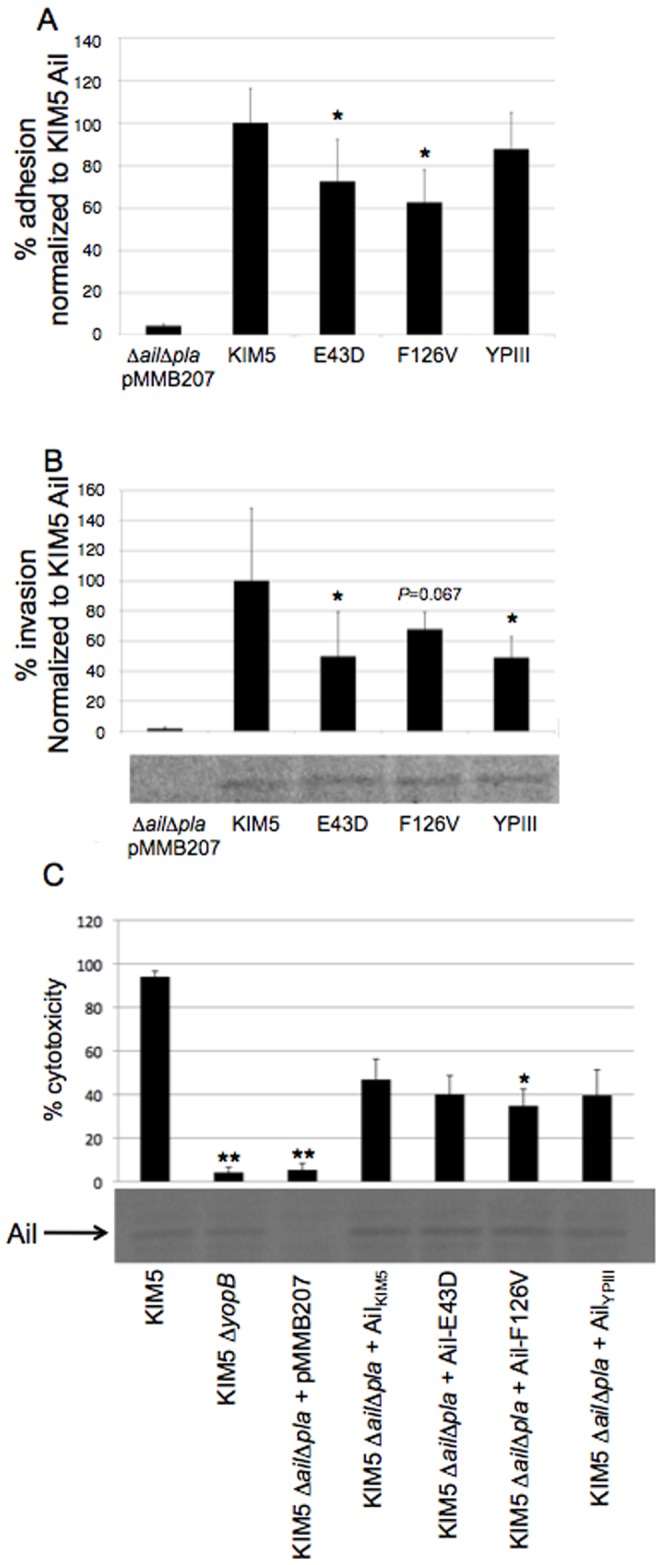
*Y. pseudotuberculosis* Ail and the *Y. pestis* Ail single mutants have defects in HEp-2 cell adhesion and invasion relative to *Y. pestis* Ail, when expressed in *Y. pestis*. A) When expressed in the KIM5 Δ*ail*Δ*pla* strain, Ail-E43D and Ail-F126V have significantly lower levels of cell adhesion to HEp2-cells than KIM5 Ail; MOI = 50 bacteria/cell. Actual adhesion by *Y. pestis* Ail was ∼6%, n = 6. B) Levels of HEp-2 cell invasion by KIM5 Δ*ail*Δ*pla* expressing Ail-E43D, Ail-F126V and YPIII Ail, were also less than that by KIM5 Ail, although the Ail-F126V result had a *P* value of 0.067, rather than 0.05, MOI = 50. Invasion refers to the number of *Y. pestis* surviving gentamicin treatment for 1 hr after a 2 hour infection of cells, compared to the bacterial inoculum over the same time period on HEp-2 cells, n = 9. Ail expression levels were assessed by Coomassie gel staining. Actual invasion by *Y. pestis* Ail was ∼1.0%. C) HEp-2 cells were infected with KIM5 Δ*ail*Δ*pla* expressing the various Ail derivatives at an MOI of 10, to determine Ail-mediated cytotoxicity. After 4 hours of infection, cells were fixed and stained with Geimsa to show shrunken, round, darker cells, indicative of Yop-mediated cytotoxicity. Cells were counted and the percent of cytotoxic cells were calculated, n = 12. Again, Ail expression was assessed by Coomassie gel staining. *P<0.05, **P<10^−11^.

Each derivative was expressed in a *Y. pestis* KIM5 Δ*ail*Δ*pla* strain. Like our findings in *E. coli*, there was a significant difference in adhesion activity for *Y. pestis* Ail, as compared to Ail-E43D and Ail-F126V, although the trend toward reduced adhesion for YPIII Ail did not reach statistical significance (Fig. 5A) and adhesion defects were not as dramatic as in E. coli (Fig. 3A). Background adhesion was low in this strain (∼0.25% in the presence of empty vector pMMB207) as it is deleted for both major *Y. pestis* adhesions, Ail and Pla [34]. Invasion activities by the various Ail derivatives were also significantly different in *Y. pestis* KIM5 Δ*ail*Δ*pla* (Fig. 5B), although one clone, Ail-F126V, did not reach statistical significance (*P* = 0.067). *pla* encodes plasminogen activator, the major mediator of cell invasion in *Y. pestis* [6], [44]. As with adhesion, in *Y. pestis* the invasion defects for the various Ail derivatives were not as drastic as in *E. coli* (Fig. 3C).

Perhaps reflecting the modest defects of *Y. pseudotuberculosis* forms of Ail on adhesion and invasion in *Y. pestis*, the ability of Ail to facilitate Type III secretion of Yop effector proteins into host cells was no different in *Y. pestis* Δ*ail*Δ*pla* expressing *Y. pestis* Ail, Ail-E43D or YPIII Ail, and only a modest 25% reduction in Yop delivery was observed with Ail-F126V (Fig. 5C). While the KIM5 Δ*ail*Δ*pla* derivative led to only 5% Yop-mediated cytotoxicity, comparable to the Δ*yopB* derivative, expression of all four Ail derivatives tested resulted in 35–45% cytotoxicity in 4 hours (Fig. 5C). YopB is part of the YopB/D translocon complex of the T3SS, and is required for Yop delivery [7].

### Ail Lacks Adhesion or Invasion Activity when Expressed in *Y. pseudotuberculosis*

**Figure 6:**
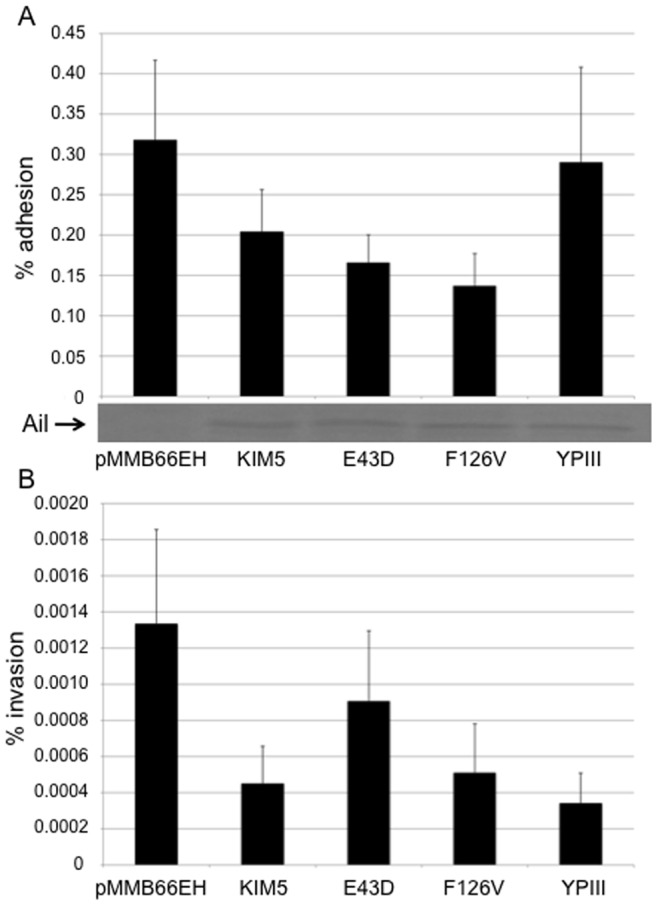
Ail has no adhesive or invasive activity when express in *Y. pseudotuberculosis*. Various Ail derivatives were expressed in the *Y. pseudotuberculosis* strain YP18 P- that lacks expression of numerous *Y. pseudotuberculosis* adhesins including: invasin, pH 6 antigen, YadA and Ail. All four Ail derivatives, including KIM5 Ail, failed to conferred adhesive (A) or invasive (B) ability on YP18 P-. YP18 P- strains were induced overnight with 0.5 mM IPTG to induce Ail expression and then diluted 1∶50 into fresh media with 0.5 mM IPTG for 3 hrs prior to infecting cells. Ail levels were assessed by Coomassie gel straining. Assays were performed at an MOI of 50 bacterial/cell, n = 6.

We next assessed the adhesion and invasion activity of each Ail derivative expressed in *Y. pseudotuberculosis*, which has an O-antigen-containing LPS [45]. The strain used was a YPIII derivative lacking Ail, invasin, pH 6 antigen and the YadA-encoding virulence plasmid, pYV (YP18 P-; *ail::Cm^R^*, *inv::Tet^R^*, *psaABC::Kan^R^*, pYV-). Expression of KIM5 Ail or the other Ail derivatives in strain YP18 P- gave no adhesion or invasion above background levels observed in the presence of the empty expression vector, pMMB66EH (Fig. 6), even though the proteins were expressed (Fig. 6). It is notable that the levels of YP18 P- adhesion and invasion in the presence of various forms of Ail is very low, 0.15–0.3% and 0.0004–0.0012%, respectively. This compares with 6% adhesion and 1% invasion by KIM5 Ail when expressed in *Y. pestis*. Thus, the context of the outer membrane environment in which Ail is expressed has an impact on the ability of Ail to function as an adhesin and direct invasion of host cells.

### A T7I Mutation in *Y. pseudotuberculosis* Ail Renders the Protein Unstable

**Figure 7:**
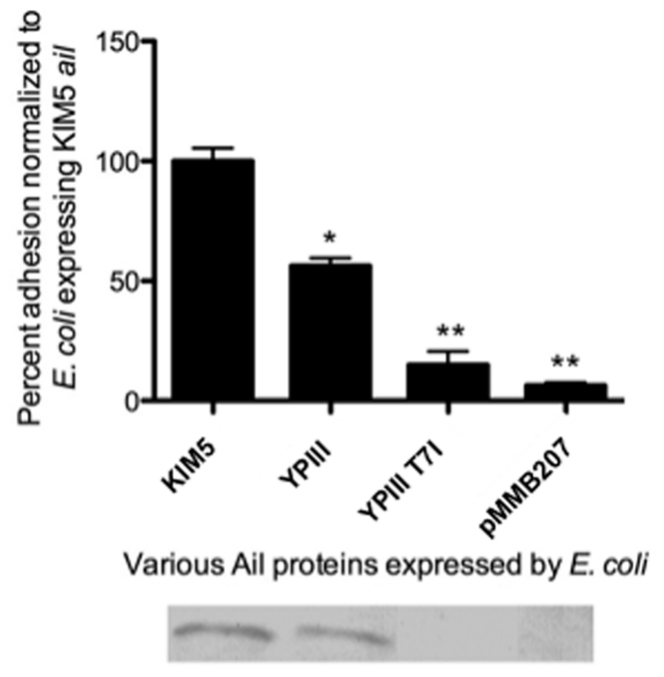
*Y. pseudotuberculosis* Ail containing the T7I mutation is unstable. HEp-2 cells were infected with *E. coli* AAEC185 expressing the *Y. pestis* KIM5 Ail, *Y. pseudotuberculosis* Ail, and *Y. pseudotuberculosis* Ail harboring a T7I substitution. Percent adhesion was calculated by dividing the number of cell-associated CFU by the total number of bacteria in the well and multiplying by 100. HEp-2 adhesion average = 4.7%. Ail expression levels were determined in whole cell extracts separated by SDS-PAGE followed by anti-Ail Western blotting. Data are from two independent experiments performed in triplicate (n = 6).

To address differences between our results and a previous report on *Y. pseudotuberculosis* Ail expressed in *E. coli*, we also generated the T7I mutation in the *Y. pseudotuberculosis* YPIII background. This Ail derivative has three amino acid changes relative to *Y. pestis* Ail (T7I, E43D, and F126V), and is the reported YPIII sequence from the previous study [30]. This version of Ail was expressed in *E. coli* and tested for adhesion to HEp-2 cells. *Y. pseudotuberculosis* YPIII Ail harboring the T7I mutation was unstable and conferred no adhesive function (Fig. 7). Therefore, the T7I mutation likely explains why a previous report found no adhesion activity for *Y. pseudotuberculosis* Ail [30]. We did not find the T7I substitution in two *Y. pseudotuberculosis* strains from which we sequenced *ail*, one of which is the same strain (YPIII) reported previously to contain the T7I substitution. Nor was the T7I substitution present in three other *Y. pseudotubercul*osis sequenced strains (Fig. 1).

## Discussion

The Ail protein of pathogenic Yersinia species is an outer membrane protein comprised of eight transmembrane ß-strands and four extracellular loops of 10–21 amino acids available for substrate binding [13], [29], [46]. Those substrates include the host cell ECM proteins, fibronectin and laminin [12], [13], [35], as well as the complement regulatory proteins C4bp and factor H [26], [27], [28], [31], [32]. While the Ail extracellular loops of *Y. enterocolitica* have diverged significantly from *Y. pestis* and *Y. pseudotuberculosis* (55% identity over the four loops), the *Y. pestis* and *Y. pseudotuberculosis* Ail molecules differ in zero, one or two amino acid residues depending on the strain (Fig. 1). Based on a previous report that *Y. pseudotuberculosis* Ail from strain YPIII is unable to mediate *E. coli* binding to host cells [30], we sought to identify residues important for the interaction of *Y. pestis* Ail with host cells. Two mutants, Ail-E43D and Ail-F126V, derived from *Y. pestis* Ail, and *Y. pseudotuberculosis* YPIII Ail (Ail-E43D/F126V, relative to *Y. pestis* Ail) were analyzed for cell binding and cell invasion capacity in *E. coli*. We found the Ail-E43D mutation is more defective than Ail-F126V for host cell binding and invasion, although both mutations affected these activities (Fig. 3). When expressed in *Y. pestis*, Ail-E43D, Ail-F126V and YPIII Ail also had defects in cell binding and invasion activities, albeit slightly less defective than what was found in *E. coli* (Figs. 3 and 5). Since *Y. pestis* contains an unusually short LPS (no O-antigen [47]), we assessed the cell-binding activity of KIM5 Ail, Ail-E43D, Ail-F126V and YPIII Ail in the *Y. pseudotuberculosis* YPIII strain background, which has a full complement of LPS with O-antigen. In a YPIII strain background lacking other adhesins (YP18 P-; *ail::Cm^R^*, *inv::Tet^R^*, *psaABC::Kan^R^*, pYV-), none of the Ail derivatives, including KIM5 Ail were able to mediated cell adhesion or invasion (Fig. 6). Previous studies have demonstrated that the proteolytic and adhesive activities of another *Y. pestis* outer membrane protein, the protease/adhesin plasminogen activator (Pla), is inhibited by the longer LPS present in *Y. pseudotuberculosis* [48]. Thus, Ail and Pla may share a preference for certain outer membrane environments for function. It is clear that some activities of Ail are maintained in *Y. pseudotuberculosis* as it has been demonstrated that Ail mediates serum resistance in *Y. pseudotuberculosis* [30], [31]. However, serum resistance protects against host complement components that must reach the bacterial outer membrane to mediate their effects. Thus, Ail would have access to such host proteins, whereas access to extracellular matrix proteins and host cell receptors by the relatively short Ail protein may be sterically occluded by the lengthy LPS in *Y. pseudotuberculosis*. It remains possible that under certain conditions at 37°C *in vivo Y. pseudotuberculosis* O-antigen may be down-regulated, revealing Ail for cell-binding and matrix-binding activity [45], [49]. Additionally, wild-type *Y. pseudotuberculosis* has a number of other adhesins to facilitate cell binding, including YadA, invasin and pH 6 antigen.

We previously reported that the extracellular matrix component, fibronectin, is a host cell substrate for *Y. pestis* Ail [12]. We hypothesized the defect observed in *E. coli* host cell binding by the single mutants as well as *Y. pseudotuberculosis* YPIII Ail, was due a lower affinity for fibronectin. Upon binding to immobilized fibronectin Ail-E43D, Ail-F126V, and *Y. pseudotuberculosis* Ail had slightly reduced binding to fibronectin as compared to *Y. pestis* Ail at 10 µg/ml and 20 µg/ml fibronectin (Fig. 4; although the defect for the E43D mutant did not reach statistical significance; p = 0.13 at 10 µg/ml and p = 0.08 at 20 µg/ml.) It is unclear why defects in binding to purified fibronectin do not precisely reflect defects in host cell binding, although we have noted previously that Ail recognizes cell-deposited and assembled fibronectin matrices somewhat differently than immobilized purified fibronectin [12].

*Y. pseudotuberculosis* Ail was previously reported to lack adhesion and invasion ability to HEp-2 cells [30]. The previous experiments were done similarly to our experiments in a heterologous *E. coli* expression system. The previously published YPIII *Y. pseudotuberculosis* Ail sequence from that study contained an additional T7I substitution that we did not observe in either of our *Y. pseudotuberculosis* strains, YPIII or IP2666, or in other *Y. pseudotuberculosis* sequenced strains (Fig. 1). Furthermore, upon generation of the *Y. pseudotuberculosis* Ail-T7I substitution, we found this particular protein (Ail-T7I/E43D/F126V) was unstable, potentially explaining the previous findings (Fig. 7).

Studies presented here indicate that various versions of Ail expressed by *Y. pseudotuberculosis* strains do have adhesion and invasion activity, but the adhesion and invasion capacity is less than *Y. pestis* Ail when expressed in *E. coli* (Fig. 3) or *Y. pestis* (Fig. 5). Future studies will be aimed at identifying residues that when substituted eliminate Ail adhesive activity in the natural context of *Y. pestis*.

## Supporting Information

Table S1 **Strains and plasmids used in this study.**
(PDF)
