# Supplementary material for: Ail Proteins of Yersinia pestis and Y. pseudotuberculosis Have Different Cell Binding and Invasion Activities
Source: PLoS One. 2013 Dec 27;8(12):e83621. doi: 10.1371/journal.pone.0083621 (PMC3873954; doi:10.1371/journal.pone.0083621)
Supplement: Table S1 — Strains and plasmids used in this study. (PDF) [file pone.0083621.s001.pdf]

Table S1: Strains and plasmids used in this study

**Strains or Plasmid**	**Genotype or Features**	**Reference or Source**
**Strains**		
E. coli		
AAEC185	*supE44 hsdR17 mcrA mcrB endA1 thi-1 ΔfimB-fimH ΔrecA*	41
DH5α	*supE44 ΔlacU169(F80lacZDM15) hsdR17 recA1 endA1 gyrA96 thi-1 relA1*	Laboratory collection
Y. pestis		
KIM D27 (referred to as KIM5)	Parental strain, *pgm–*	Dr. Melanie Marketon
KIM5 D27Δ*ail*Δ*pla*	*pgm– ΔailΔpla*	This study
KIM5 D27Δ3	*pgm– ΔailΔplaΔpsaA*	This study
KIM5 D27 Δ*yopB*	*pgm– ΔyopB*	This study
Y. pseudotuberculosis		
IP2666	Wild-type strain	Dr. James Bliska
YPIII	Wild-type strain	Drs. James Bliska and Ralph Isberg
YP18	YPIII *inv:tet*^{R}*, psaABC::kan*^{R}*, ail::Cm*^{R}	Dr. Petra Dersch
YP18 pYV-	YPIII *inv:tet*^{R}*, psaABC::kan*^{R}*, ail::cm*^{R} pYV- (*yadA*-, plasmid cured)	This study
**Plasmids**		
pMMB207	IPTG-inducible expression plasmid, Cm^R	38
pMMB207-*ail* KIM5	Ail$_{KIM5}$-expressing plasmid	6
pMMB207-*ail*-E43D	Ail-E43D-expressing plasmid	This study
pMMB207-*ail*-F126V	Ail-F126V-expressing plasmid	This study
pMMB207-*ail* YPIII (E43D/F126V)	Ail$_{YPIII}$-expressing plasmid	This study
pMMB207-*ail*-T7I/E43D/F126V	Ail-T7I/E43D/F126V-expressing plasmid	This study
pMMB66EH	IPTG-inducible expression plasmid, Amp^R	39
pMMB66EH-*ail* KIM5	Ail$_{KIM5}$-expressing plasmid	This study
pMMB66EH-*ail* E43D	Ail-E43D-expressing plasmid	This study
pMMB66EH-*ail* F126V	Ail-F126V-expressing plasmid	This study
pMMB66EH-*ail* YPIII	Ail$_{YPIII}$-expressing plasmid	This study
